# Social Media for Epidemiological Journals

**DOI:** 10.2188/jea.JE20230006

**Published:** 2023-05-05

**Authors:** Soichiro Saeki

**Affiliations:** Center Hospital of the National Center for Global Health and Medicine, Tokyo, Japan

**Keywords:** social networking services, academic journals, health promotion, COVID-19, bibliometrics

The significance of the internet as a source of medical information has been widely acknowledged,^1^ and its importance has progressively grown over the years. Many individuals source various information online, with researchers and medical practitioners being no exception.

Notably, during the novel coronavirus disease, 2019 (COVID-19) pandemic, social networking services (SNS) have proven to be a vital instrument for distributing health-related information, as internet-based communication mitigates the risks of face-to-face interactions and the potential spread of infection.^2^^,^^3^ Additionally, society has come to rely on social media as an information-gathering tool in the event of newly emerging pandemics,^4^ such as the ongoing COVID-19 pandemic.^5^ A plethora of information was distributed via various SNS,^5^ including misinformation, leading to an “infodemic”.^6^ This necessitated the need for health experts to swiftly and accurately disseminate information to overcome such challenges.^7^

In light of this, we wholeheartedly embrace the introduction of the new Twitter account by the *Journal of Epidemiology*,^8^ which was launched in July 2022. Not only will such dissemination of information contribute to the advancement of public health, but it also has the potential to enhance the *Journal*’s presence in the medical community. Several studies have shown that promoting manuscripts on Twitter increases article views and citations in various fields, such as stroke,^9^ emergency medicine,^10^^,^^11^ and other disciplines,^12^^–^^14^ which can benefit the authors by increasing their research’s visibility to potential readers. Twitter can be used to interact directly with authors, reviewers, and readers, facilitating more efficient communication and establishing new connections with other academic journals and researchers. The account can also be used to share related news, upcoming events, and academic meetings, which can also increase academic discourse among researchers. Indeed, several other epidemiology journals actively share content on Twitter (Table 1).

**Table 1.** Demographics of Twitter accounts of epidemiological journals

Journal	Twitter account	Account created	Followers
*J Epidemiol Community Health*	@JECH_BMJ	May, 2010	4,035
*Epidemiology*	@EpidemiologyLWW	December, 2010	10,710
*Am J Epidemiology*	@AmJEpi	August, 2014	17,554
*Int J Epidemiol*	@IntJEpidemiol	October, 2014	7,228
*J Clin Epidemiol*	@JClinEpi	December, 2014	8,645
*Cancer Epidemiol Biomarkers Prev*	@CEBP_AACR	July, 2018	1,243
*Ann Epidemiol*	@AnnalsOfEpi	September, 2019	139
*J Epidemiol*	@J_Epidemi	July, 2022	202

Twitter accounts were aligned in the order when the account was created. Journal names are indicated with MedAbbr from PubMed. Followers of each account was counted on 14:00, January 23, 2023 (Japan Standard Time). Twitter accounts were selected under the discretion of the author.

However, several challenges can be observed. First, in order to fully realize the potential benefits of promotion through Twitter, the manner in which it is used would be crucial. Some studies have argued that an ordinary tweet from an official Twitter account does not serve to increase visibility,^15^ and proposed that interactions between potential researchers are essential.^16^ Second, the potential benefits of Twitter for official accounts in epidemiology have not been fully studied. A search on PubMed with the query “((epidemiology) AND (twitter) AND (journal)) AND (citation)” on January 12, 2022, revealed eight potential studies, and only one^15^ was related to the promotion of journal manuscripts in the field of public health. Thus, the *Journal* is poised to serve as a pioneer in the usage of Twitter in the field of epidemiology.

Overall, we are thrilled to have a new Twitter account launched by the *Journal of Epidemiology*. We fully support this ambitious approach by the editorial team, and as potential authors and readers of the *Journal*, we are eager to play a role in utilizing the new platform to conduct new discussions and disseminate novel information based on the evidence presented in the *Journal*.
